# SOX4 expression is associated with treatment failure and chemoradioresistance in oral squamous cell carcinoma

**DOI:** 10.1186/s12885-015-1875-8

**Published:** 2015-11-10

**Authors:** Tae Mi Yoon, Sun-Ae Kim, Wan Seok Cho, Dong Hoon Lee, Joon Kyoo Lee, Young-Lan Park, Kyung-Hwa Lee, Jae Hyuk Lee, Sun-Seog Kweon, Ik-Joo Chung, Sang Chul Lim, Young-Eun Joo

**Affiliations:** 1 Departments of Otorhinolaryngology-Head and Neck Surgery, Chonnam National University Medical School and Hwasun Hospital, 8 Hak-Dong, Dong-Ku, Gwangju 501-757 South Korea; 2 Departments of Internal Medicine, Chonnam National University Medical School and Hwasun Hospital, 8 Hak-Dong, Dong-Ku, Gwangju 501-757 South Korea; 3 Departments of Pathology, Chonnam National University Medical School and Hwasun Hospital, 8 Hak-Dong, Dong-Ku, Gwangju 501-757 South Korea; 4 Departments of Preventive Medicine, Chonnam National University Medical School and Hwasun Hospital, 8 Hak-Dong, Dong-Ku, Gwangju 501-757 South Korea

**Keywords:** SOX4 protein, Radioresistance, Apoptosis, Molecular targeted therapy, Oral cancer, Head and neck squamous cell carcinoma

## Abstract

**Background:**

In humans, sex-determining region-Y (SRY) related high-mobility-group box 4 (SOX4) is linked to development and tumorigenesis. SOX4 is over-expressed in several cancers and has prognostic significance. This study evaluated whether SOX4 affects oncogenic behavior and chemoradiotherapy response in head and neck squamous cell carcinoma (HNSCC) cells, and documented the relationship between its expression and prognosis in oral squamous cell carcinoma (OSCC).

**Methods:**

We used small interfering RNA in HNSCC cells to evaluate the effect of SOX4 on cell proliferation, apoptosis, chemoradiation-induced apoptosis, invasion, and migration. SOX4 expression in OSCC tissues was investigated by immunohistochemistry.

**Results:**

*SOX4* knockdown (KO) decreased cell proliferation and induced apoptosis by activating caspases-3 and −7, and poly-ADP ribose polymerase and suppressing X-linked inhibitor of apoptosis protein in HNSCC cells; it also enhanced radiation/cisplatin-induced apoptosis; and suppressed tumor cell invasion and migration. Immunostaining showed SOX4 protein was significantly increased in OSCC tissues compared with adjacent normal mucosa. SOX4 expression was observed in 51.8 % of 85 OSCC tissues, and was significantly correlated with treatment failure (*P* = 0.032) and shorter overall survival (*P* = 0.036) in patients with OSCC.

**Conclusions:**

SOX4 may contribute to oncogenic phenotypes of HNSCC cells by promoting cell survival and causing chemoradioresistance. It could be a potential prognostic marker for OSCC.

**Electronic supplementary material:**

The online version of this article (doi:10.1186/s12885-015-1875-8) contains supplementary material, which is available to authorized users.

## Background

Oral cavity cancer accounts for approximately 28 % of all head and neck cancers [1]. Squamous cell carcinomas represent about 90 % of oral cavity cancer. Oral squamous cell carcinoma (OSCC) is the sixth most prevalent malignancy worldwide and the third most common cancer in developing nations [1]. Surgery, radiation therapy, chemotherapy or combinations of these modalities are standard options for managing OSCC; therapeutic strategies are based on disease stage [2]. As advanced-stage OSCC has a poor prognosis [1], understanding the molecular and biological changes of its progression is critical to development of more effective therapies.

In humans, the sex-determining region Y (SRY) related high-mobility-group (HMG) box family—also called the SOX family—includes 20 highly conserved transcription factors that affect diverse developmental processes [3]. SOX4 is essential to endocardial development and lymphocyte differentiation [3, 4]. Reportedly, SOX4 expression results in alterations of oncogenic phenotypes, including inhibition of apoptosis, cell-cycle progression and irradiation-induced apoptosis, and promotion of epithelial to mesenchymal transition in a variety of cancer cells [5–12]. SOX4 expression has also been reported to be highly expressed in various cancer tissues [5–14]. However, its role in tumor progression and clinical outcomes is unclear and has shown certain contradictions in different cancers. High SOX4 expression has been associated with better prognosis for patients with hepatocelluar carcinoma, medulloblastoma, and bladder cancer [5, 13, 14], but with shorter survival in prostate cancer, gastric cancer, and colon cancer [7, 9, 10]. Thus, SOX4 might exert different effects depending on tumor cell types and context.

Little is known about the molecular and prognostic significance of SOX4 in OSCC, although one report correlated SOX4 expression with OSCC tumor stage [12]. In the present study, we investigated whether SOX4 affects tumor cell behaviors such as cell proliferation, apoptosis, invasion, migration, and chemoradiation-induced apoptosis in head and neck squamous cell carcinoma (HNSCC) cells to validate its potential as a novel molecular target. We also assessed its prognostic value in OSCC.

## Methods

### Cell culture and transfection

The HNSCC cell lines (PCI 50 and SNU 1041) were kindly provided by Dr. Sung MW (Seoul National University, Seoul, South Korea). The normal immortalized human keratinocyte cell line (HaCaT) was purchased from the American Type Culture Collection (Manassas, VA, USA). Cell lines were cultured in DMEM or RPMI1640 (Hyclone, Logan, UT, USA) supplemented with 10 % fetal bovine serum (Hyclone) in a humidified atmosphere of 5 % CO_2_ at 37 °C. For transfection, cells were seeded on 6-well plates at 2 × 10^5^ cells per well at the time of transfection. Small interfering RNA (siRNA) was used to knock down endogenous *SOX4* gene expression in HNSCC cells. Cells were transfected with *SOX4*-specific siRNA (Bioneer, Daejeon, Korea) or negative control siRNA (Qiagen, Valencia, CA, USA) using Lipofectamine™ 2000 (Invitrogen, Carlsbad, CA, USA) for 48 h. SOX4 knockdown (*SOX4*-KO) was checked by reverse transcription-polymerase chain reaction (RT-PCR) and western blotting.

### RNA isolation and RT-PCR

The total RNA from cells was extracted using Trizol reagent (Invitrogen), reverse transcribed, and amplified using specific primers for *SOX4* and glyceraldehyde 3-phosphate dehydrogenase (*GAPDH*), as previously described [15]. The extracted RNA loading was performed to verify the RNA integrity and double band (18S and 28S) was detected (Additional file 1). Primer sequences were: *SOX4*: 5′-GCA CAT GGC TGA CTA CCCC −3′/ 5′-GCC TTGTAC AGC GAG TGG TG-3′; and *GAPDH*: 5′-ACC ACA GTC CAT GCC ATC AC-3′/ 5′-TCC ACC ACC CTG TTG CTG TA-3′. PCR products were separated by electrophoresis on a 1 % agarose gel containing ethidium bromide. The signals were quantified by densitometric analysis using the Labworks Image Acquistion (UVP, Upland, CA).

### Protein isolation and western blot analysis

Cells were lysed in RIPA buffer. Resolved proteins were electrophoretically transferred to polyvinylidene fluoride membranes. Specific proteins were sequentially blotted with primary antibodies: SOX4 (Catalogue# ab80261, Abcam, Cambridge, Mass, USA), cleaved caspase-3, cleaved caspase-7, cleaved poly-ADP ribose polymerase (PARP; Cell Signaling Technology, Danvers, MA, USA), X-linked inhibitor of apoptosis protein (XIAP) and polyclonal anti-GAPDH (Santa Cruz Biotechnology, CA, USA). Each membrane was incubated with anti-rabbit or anti-mouse HRP-conjugated secondary antibody (Santa Cruz Biotechnology). Immunoreactive proteins were visualized on the enhanced chemiluminescence detection system HRP substrate (Millipore, Billerica, MA, USA). The immunoreactive bands were quantified by densitometric analysis using the luminescent image analyzer LAS-4000.

### Cell proliferation assay

Cells were seeded in a 96-well plate (5 × 10^3^ cells/well), and were transfected with SOX4 siRNA and negative control siRNA the next day. After incubation for 48 h, cell proliferation and viability were measured using the EZ-CyTox (tetrazolium salts, WST-1) cell viability assay kit (Daeil Lab Inc, Seoul, South Korea). After adding WST-1 reagent for 1–2 h at 37 °C, absorbance at 460 nm was determined using a microplate reader (Infinite M200; Tecan, Austria GmbH, Austria) with Magellan V6 data analysis software (Tecan). Triplicate wells were used for experimental conditions and all experiments were repeated at least three times.

### Apoptosis assay

Apoptosis was determined by an Annexin V-fluorescein isothiocyanate (FITC) assay. Forty-eight hours after transfection, cells transfected with *SOX4* siRNA or negative control siRNA were collected using trypsin, washed twice in phosphate buffered saline (PBS), and re-suspended in binding buffer (BD Biosciences, San Diego, CA, USA). Annexin V-FITC and 7-amino-actinomycin D (7-AAD; BD Biosciences) were added to the cells, which were incubated in the dark for 15 min, then re-suspended in 400 ml of binding buffer. Cells were analyzed using a FACSCalibur flow cytometer (Becton Dickinson, San Jose, CA). Data analysis was performed using standard Cell Quest software (Becton Dickinson).

### Cell irradiation and Cisplatin treatment

Cells were treated with γ-irradiation at a single dose of 5 Gy (^137^Cs, 2.875 Gy/min) using a Gammacell irradiator (Gammacell, Otawa, Canada) [16, 17]. Cells were treated with cisplatin at 10 μg/ml (Pharmachemie BV, New York, USA) for 24 h at 37 °C.

### Cell invasion assay

Cell invasion ability was measured by the number of cells that invaded through a transwell invasion apparatus with 8.0-μm pores (Costar, Cambridge, UK). Living cells transfected with *SOX4* siRNA or negative control siRNA were seeded at 3 × 10^5^ cells in 120 μl of a 0.2 % bovine serum albumin (BSA) suspension in the upper chamber. We then loaded 400 μl of 0.2 % BSA containing 7-μg/ml fibronectin (Calbiochem, La Jolla, CA, USA) into the lower chamber as the chemoattractant. After incubation for 24 h, cells that had moved to the bottom Transwell surface were stained with Diff Quik solution (Sysmex, Kobe, Japan) and calculated in five random squares in the microscopic field of view. Results are shown as mean ± standard error of the number of cells/field in three individual experiments.

### Cell migration assay (wound healing assay)

Cells transfected with *SOX4* siRNA or negative control siRNA were seeded in each well of Culture-Inserts (Ibidi, Bonn, Germany) at 1.5 × 10^5^ cells/well. After incubation for 24 h, each insert was detached and the progression of cell migration was ascertained by photography at 0, 4, 8, 12, and 24 h, using an inverted microscope. Distances between gaps were normalized to 1 cm after capture of three random sites.

### Patients and tumor specimens

To evaluate SOX4 protein expression, paraffin-embedded tissue sections were collected from 95 patients who had undergone diagnostic biopsy or definitive surgery for OSCC at Chonnam National University Hwasun Hospital (Jeonnam, Korea) between May 2004 and June 2013. None of the collected tissues were obtained after radiotherapy and/or chemotherapy. Ten patients were excluded, because of follow-up loss or palliative treatment intent. Of the 85 remaining patients, 82 patients were treated with definitive surgery with/without adjuvant radiotherapy or cisplatin-based concurrent chemoradiotherapy (CRT). Three patients, who refused surgery, were treated with induction chemotherapy, followed by cisplatin-based concurrent CRT with curative intent. Patients with locoregional recurrence after primary treatment underwent salvage surgery or CRT. Of 85 patients in our study, 50 (58.8 %) underwent chemotherapy and/or radiotherapy. Treatment failure was defined as disease with inoperable locoregional progression or distant metastasis, even through salvage treatment. Patients provided the written informed consents for the surgical procedures, as well as for the use of resected tissue specimens. Patients’ clinicopathologic characteristics were reviewed in hospital records. Tumors were staged according to the seventh edition of the American Joint Committee on Cancer staging system [18]. Survival was measured from the date of starting treatment to the date of death or date last seen. This study was approved by the Institutional Review Board of Chonnam National University Hwasun Hospital (CNUHH-2015-028).

### Immunohistochemistry

Tissue processing and immunohistochemical analysis were performed as previously described [15]. The tissues were incubated with polyclonal rabbit anti-human SOX4 (Abcam). Immunohistochemsitry was performed in five batches, averaging 18 samples, with one positive and one negative control per batch. Negative controls were treated similarly, except that primary antibodies were omitted.

Two independent observers interpreted SOX4 staining of specimens with no knowledge of the clinical information. Intensity was scored as follows: 0, no staning of tumor cells; 1+, weak to comparable staining in cytoplasm and/or the nucleus compared to that of non-tumoral cells; 2+, readily appreciable or dark brown staining distinctly marking the tumor cell cytoplasm and/or nucleus [10]. Percentages of stained cells were scored as follows: 0: 0 %; 1: 1–25 %; 2: 26–50 %; 3: 51–75 %; and 4, >75 % [6, 7]. Final staining scores were the product of the intensity and percentage scores, with ≤4 defined as low SOX4 expression and >4 defined as high SOX4 expression. Staining scores were discordant between the two pathologists (KHL and JHL) in five cases (5/85, *κ* = 0.875), which were re-evaluated by the two pathologists, who then reached an agreement for each inconclusive sample.

### Statistical analyses

Relationships between SOX4 expression and various clinicopathologic parameters were compared using the *χ*^2^ test and Fisher’s exact test. Survival curves were calculated by the Kaplan–Meier method, and compared using the log-rank test. Experimental differences between the *SOX4*-KO group and control group were tested with the Mann–Whitney *U* test. Analyses used Statistical Package for the Social Sciences (SPSS) version 21.0 (Microcal Software Inc, Chicago, IL, USA). *P* < 0.05 was considered significant.

## Results

### *SOX4-KO* suppresses tumorigenic activities in HNSCC cells

**Fig. 1:**
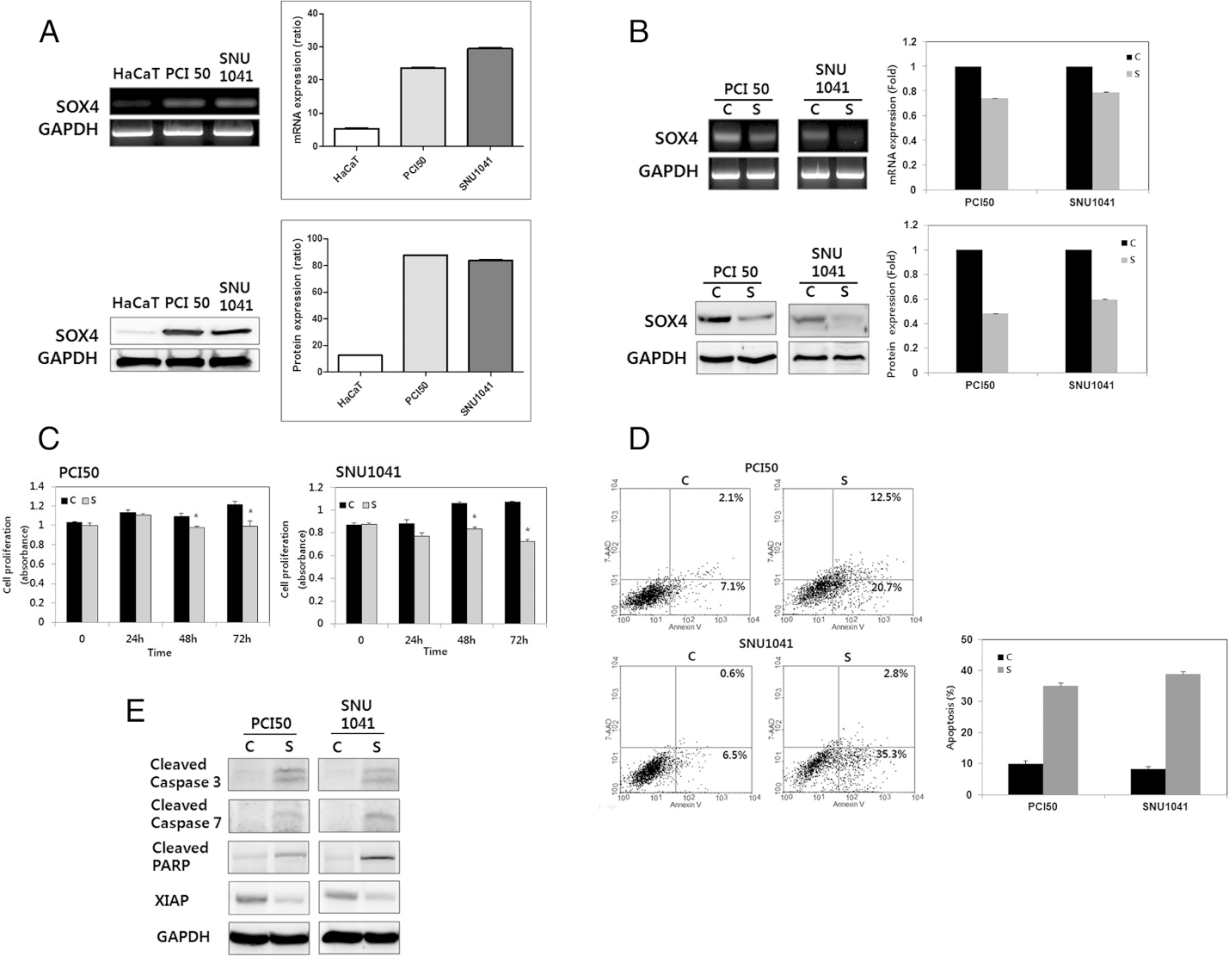
Effect of SOX4 knockdown on cell proliferation and apoptosis in head and neck squamous cell carcinoma (HNSCC) cells. **a** SOX4 protein and mRNA were well-expressed in HNSCC cells, PCI50 and SNU1041 cells, but not in normal keratinocyte, HaCaT cells. **b** SOX4 protein and mRNA expressions were reduced by *SOX4* siRNA (*SOX4*-KO) in PCI50 and SNU 1041 cells compared with negative control siRNA. **c** Absorbance indicates that proliferation was decreased in *SOX4*-KO PCI50 and SNU1041 cells compared with negative control cells (mean ± SE, ^*^P < 0.05; experiments were run in triplicate). **d** *SOX4*-KO PCI50 and SNU1041 cells displayed more apoptosis than in control cells. **e** Levels of cleaved caspases-3 and −7, and cleaved poly-ADP ribose polymerase (PARP) were greater, and X-linked inhibitor of apoptosis protein (XIAP) was less, in *SOX4*-KO PCI50 and SNU1041 cells than in control cells. C: negative control siRNA transfected cells; S: *SOX4*-specific siRNA transfected cells (*SOX4*-KO)

Initially, SOX4 expression at mRNA and protein levels was evaluated in HNSCC cells and HaCaT cells. Western blot and RT-PCR showed SOX4 to be well-expressed in PCI50 and SNU1041 cells, but negligibly expressed in HaCaT cells relative to HNSCC cells (Fig. 1a). To explore the role of SOX4 on oncogenic activities and treatment response in HNSCC cells, we used siRNA to inhibit endogenous SOX4 expression in HNSCC cell lines including PCI50 and SNU1041 cells. SOX4 mRNA and protein expressions were reduced by *SOX4* siRNA in PCI50 and SNU 1041 cells compared with cells treated with negative control siRNA (Fig. 1b).

#### *SOX4-KO* decreases cell proliferation in HNSCC cells

Proliferating cells, as determined by absorbance, were significantly decreased in SOX4-KO cells at 48 h and 72 h in PCI50 and SNU1041 cells, as compared with negative control cells (*P* = 0.002; Fig. 1c).

#### *SOX4-KO* enhances apoptosis in HNSCC cells

To evaulate the effect of SOX4 on apoptosis, we used an Annexin V apoptosis assay. *SOX4*-KO PCI50 and SNU1041 cells displayed greater apoptotic rates than did control cells (Fig. 1d). The proportion of early and late apoptotic cells induced by transfection of SOX4 siRNA was greater than that induced by transfection of negative control siRNA (9.2 % *vs*. 33.2 % and 7.1 % *vs*. 38.1 %, respectively) in PCI50 and SNU1041 cells. Next, we investigated apoptosis regulatory proteins after *SOX4*-KO treatment. Levels of cleaved caspases-3 and −7, and PARP were increased, and the level of XIAP was decreased, in *SOX4*-KO PCI50 and SNU1041 cells, compared with negative control cells (Fig. 1e). These results suggest that *SOX4*-KO-induced apoptosis is associated with the modulation of apoptosis regulatory proteins such as caspases-3 and −7, PARP and XIAP.

### *SOX4-KO* suppresses the tumor cell invasion and migration in HNSCC cells

**Fig. 2:**
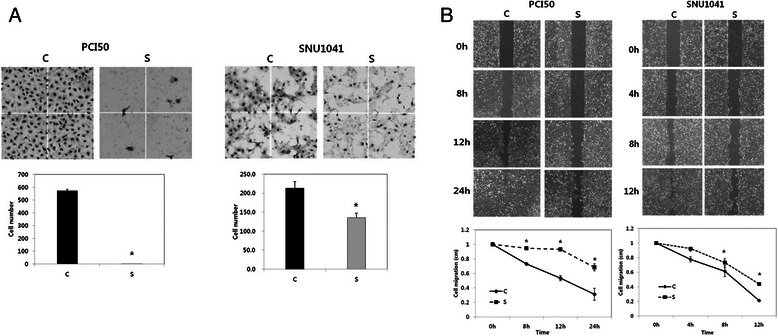
Effects of SOX4 knockdown (*SOX4*-KO) on cell invasion and migration in head and neck squamous cell carcinoma cells. **a** In the cell invasion assay, significantly fewer *SOX4*-KO PCI50 and SNU1041 cells invaded than did negative control cells. Stained invading cells were counted (bar graph; mean ± SE, experiments were run in triplicate; ^*^P < 0.05). **b** Cell migration was significantly less in *SOX4*-KO PCI50 and SNU1041 cells than in negative control cells (displayed as relative healing distances measured in three random sites). Values indicate mean ± SE for three independent experiments (^*^P < 0.05). C: negative control siRNA transfected cells; S: SOX4-specific siRNA transfected cells (*SOX4*-KO)

In the cell invasion assay, the invasiveness of *SOX4*-KO PCI50 cells and SNU1041 cells was significantly decreased compared with that of negative control cells (*P* < 0.05; Fig. 2a). In the cell migration assay, the migratory ability of *SOX4*-KO cells was significantly less than that of negative control cells at 8 h, 12 h, and 24 h in PCI50 cells, and 8 h and 12 h in SNU1041 cells (*P* < 0.05; Fig. 2b).

### *SOX4-KO* enhances radiosensitivity and cisplatin chemosensitivity in HNSCC cells

**Fig. 3:**
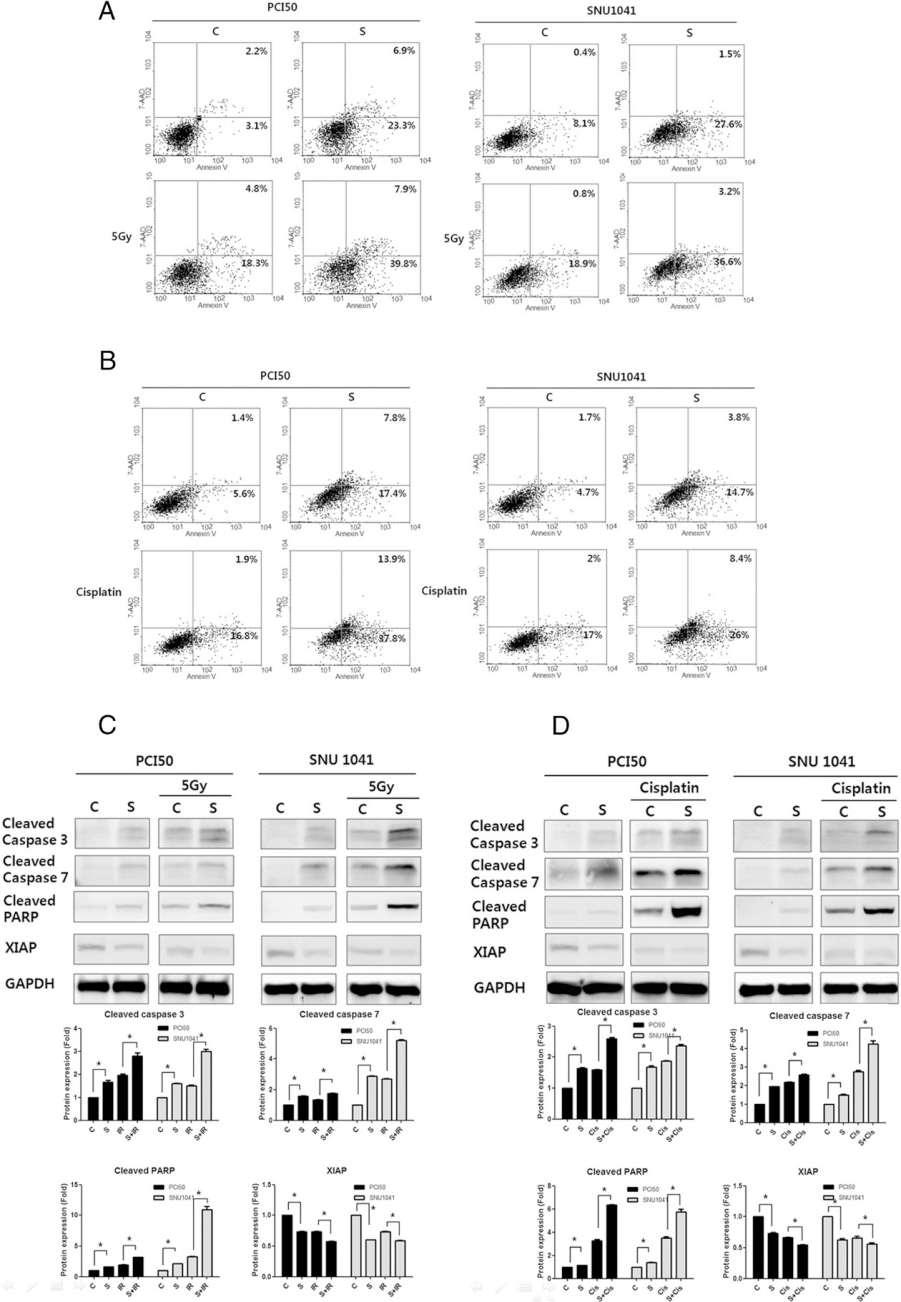
Effects of *SOX4* knockdown (*SOX4*-KO) on radiosensitivity and cisplatin chemosensitivity in head and neck squamous cell carcinoma cells. **a**, **b** Combination treatment of *SOX4*-KO with 5 Gy radiation or cisplatin resulted in significantly more apoptosis in PCI50 and SNU1041 cells than in control cells treated with 5 Gy radiation or cisplatin treatment alone. **c**, **d** *SOX4*-KO cells showed greater expression of cleaved caspases-3 and −7, and cleaved poly-ADP ribose polymerase (PARP), and less X-linked inhibitor of apoptosis protein (XIAP), than did control cells after 5 Gy radiation or cisplatin treatment (^*^P < 0.05). C: negative control siRNA transfected cells; S: *SOX4*-specific siRNA transfected cells (*SOX4*-KO); Cis: cisplatin treatment; IR: irradiation

We addressed whether *SOX4*-KO enhances cisplatin chemosensitivity and radiosensitivity by the induction of apoptosis in PCI50 and SNU1041 cells. Twenty-four hours after transfection with *SOX4* siRNA or negative control siRNA, cells were treated with 5 Gy radiation or cisplatin (10 μg/ml for 24 h). The combination of *SOX4* siRNA and radiation resulted in significantly greater apoptosis compared with radiation alone (Fig. 3a). The percentages of early and late apoptotic cells induced by *SOX4* siRNA + 5 Gy radiation were greater than those seen in negative control cells treated with 5 Gy radiation (23.1 % *vs.* 47.7 % and 19.7 % *vs.* 39.8 %, respectively) in PCI50 and SNU1041 cells. Similarly, the combination of *SOX4* siRNA + cisplatin resulted in markedly greater apoptosis compared with cells treated with cisplatin alone (Fig. 3b), with larger percentages of early and late apoptotic cells in the *SOX*4 siRNA + cisplatin-treated cells than in the negative control treated with cisplatin only (18.7 % *vs*. 51.7 % and 19.0 % *vs.* 34.4 %, respectively) in PCI50 and SNU1041 cells. Consistently, the *SOX4*-KO cells showed greater expression of cleaved caspases-3 and −7 and PARP, and less XIAP, after radiation or cisplatin treatment compared with the control cells (Fig. 3c, d). These findings suggest that the combination of *SOX4*-KO and CRT has synergistic apoptotic effects in HNSCC cells.

### SOX4 expression is associated with treatment failure and survival in OSCC

#### SOX4 expression in OSCC tissues

**Fig. 4:**
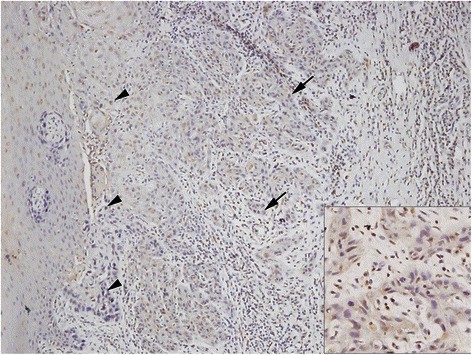
Expression of SOX4 protein in oral squamous cell carcinoma (OSCC) specimens. Immunostaining showed SOX4 protein was significantly increased in OSCC tissues (arrow) compared with adjacent normal mucosa (arrow head). (×100, ×200 in the inlet box)

The clinicopathological characteristics of the 85 OSCC patients in this study group are summarized in Table 1. The patients included 61 men and 24 women, whose mean age was 63.2 ± 12.5 years (± standard deviation), with a range of 26–87 years. Their mean follow-up period was 43.7 ± 27.7 months (range: 3.6–125.3 months). SOX4 protein expression was investigated immunohistochemically in formalin-fixed, paraffin-embedded blocks of specimens from these 85 patients. Immunostaining patterns were heterogenous, with predominantly nuclear and/or cytoplasmic immunostained SOX4 protein in tumor cells, but with weak or no staining in the normal oral mucosa (Fig. 4). Based on our criteria [6, 7, 10], 44 (51.8 %) of the 85 OSCC specimens showed high SOX4 expression

**Table 1:** Association between SOX4 expression and clinicopathological parameters in patients with oral squamous cell carcinoma

Parameters		SOX4 expression		*p*-value
	Total (*n* = 85)	Low (*n* = 41)	High (*n* = 44)	
Age (yr)				0.161
<64	41	23	18	
≥64	44	18	26	
Sex				0.492
Male	61	28	33	
Female	24	13	11	
Location				0.654
Oral tongue	60	28	32	
FOM, BM, RMT	25	13	12	
Stage				0.651
I, II	56	28	28	
III, IV	29	13	16	
T stage				0.843
T1, T2	74	36	38	
T3, T4	11	5	6	
N stage				0.327
N0	60	31	29	
N1, N2	25	10	15	
CRT				0.622
No	35	23	27	
Yes	50	18	17	
Recurrence				0.004
No	53	32	21	
Yes	32	9	23	
Treatment failure				0.032
No	59	33	26	
Yes	26	8	18	

FOM = floor of mouth; BM = buccal mucosa; RMT = retromolar trigone; CRT = chemotherapy and/or radiotherapy; *χ*^2^ test and Fisher’s exact test was used

.

#### Correlation between SOX4 expression and clinicopathologic factors in OSCC tissues

**Fig. 5:**
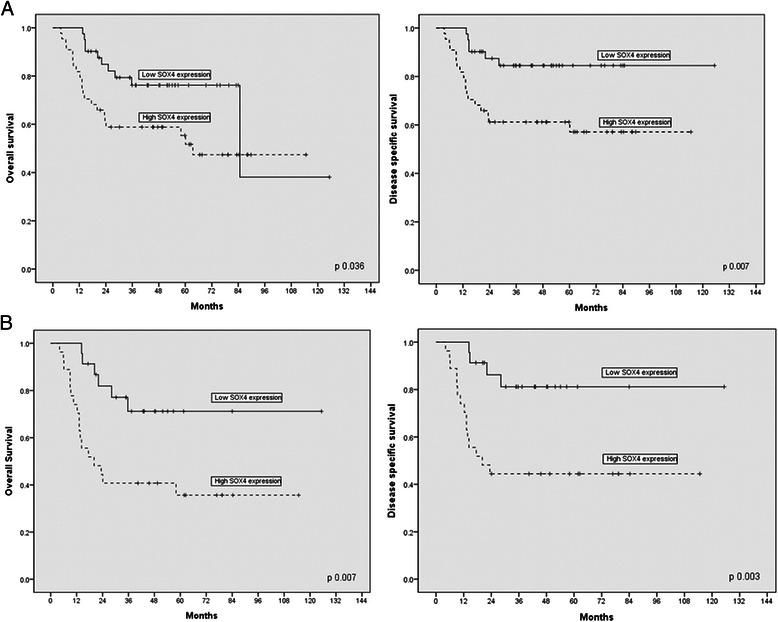
Kaplan–Meier curves of overall survival (OS) and disease specific survival (DSS) for patients with oral squamous cell carcinoma (OSCC) by SOX4 expression. **a** SOX4 expression significantly correlated with diminished OS and DSS in patients with OSCC (*n* = 85, *P* = 0.036 and *P* = 0.007, respectively). **b** SOX4 expression was associated with significantly worse OS and DSS in patients with OSCC, who were treated with chemotherapy and/or radiotherapy (*n* = 50, *P* = 0.007 and *P* = 0.003, respectively). Solid line: patients with low SOX4 expression; dotted line: patients with high SOX4 expression

To study the prognostic role of SOX4 in OSCC, we investigated the correlation between SOX4 expression and clinicopathological factors. SOX4 expression in OSCC was not associated with age, sex, location, T stage (tumor invasion), N stage or lymph node metastasis (*P* > 0.05, Table 1). However, SOX4 expression was correlated with recurrence (*P* = 0.004) and treatment failure (*P* = 0.032; Table 1). Moreover, overall survival (OS) and diease specific survival (DSS) of patients with high SOX4 expression was significantly shorter than for those with low SOX4 expression (*P* = 0.036 and *P* = 0.007, respectively; Fig. 5a). In 50 patients who were treated with chemotherapy and/or radiotherapy, patients with high SOX4 expression had significantly shorter OS and DSS than those with low SOX4 expression (*P* = 0.007 and *P* = 0.003, respectively; Fig. 5b).

## Discussion

Initiation and progression of cancer are caused by alterations in transcriptional activities, resulting in an imbalance between oncogenes and tumor suppressor genes [19]. Transcription factors are deregulated in their expression and function during tumorigenesis [19]. The SOX transcription factor family plays a key role in many developmental processes by controlling terminal differentiation of a wide variety of cell types and cell fate decisions [20]. Consequently, deregulation of several *SOX* genes have been implicated in tumorigenesis [20]. SOX4 belongs to the C subgroup of the SOX family. High SOX4 expression has been shown to affect tumor development or progression in gastric cancer, colon cancer, prostate cancer, breast cancer, lung cancer, and endometrial cancer [7–10, 21, 22]. The mechanism by which SOX4 is involved in tumor development and progression in many cancers remains unclear.

Tumorigenesis results from an imbalance between cell proliferation and cell death; most of the latter occurs through apoptosis [23]. Therefore, the primary mechanism through which SOX4 affects tumor initiation and progression may be deregulation of apoptosis. SOX4 gene encodes a protein with three distinguishable domains: an HMG box, a serine-rich region, and a glycine-rich central domain [24]. The HMG box binds DNA, whereas the central domain is a functional region for regulating apoptotic cell death [24]. Therefore, we evaluated the impact of SOX4 in cell proliferation and apoptosis in HNSCC cells. In this study, knocked-down *SOX4* induced apoptosis and suppressed cell proliferation, which indicates that SOX4 supresses apoptosis in HNSCC cells. Additionally, *SOX4*-KO-induced apoptosis was associated with the modulation of apoptosis-related proteins such as caspases-3 and −7, PARP, and XIAP. Our results concord with reports in which *SOX4*-KO in adenoid cystic carcinoma and prostate cancer cells induced apoptosis [6, 7, 25]. Therefore, SOX4 exerts its anti-apoptotic function by directly inhibiting caspase activities and up-regulation of anti-apoptotic proteins, thus contributing to tumorigenesis in HNSCC.

Second, the anti-apoptotic function of SOX4 apparently causes resistance to anti-cancer treatement such as CRT. Cancer cells are often characterized by increased resistance to apoptosis [26]. Overcoming apoptotic resistance is important to improve response to tumor treatments, especially CRT. In the present study, knocked-down *SOX4* enhanced radiation- or cisplatin-induced apoptosis in HNSCC cells, which were further supported by elevated levels of cleaved caspases-3 and −7, and PARP in *SOX4*-KO HNSCC cells after radiation or cisplatin treatment. These results suggest that SOX4 inhibits radiation- or cisplatin- induced apoptosis, and contributes to CRT resistance in HNSCC cells. These findings are very important because CRT is used as primary or adjuvant treatment for locally advanced HNSCC (including OSCC), and CRT response is accepted as an important prognostic factor [2, 27]. Our findings suggest that SOX4 can serve as a specific predictor for CRT response in HNSCC. Furthermore, a therapy in which SOX4 is targeted in combination with CRT might overcome apoptotic resistance and improve response in HNSCC.

Third, SOX4 appears to aggravate cell invasiveness and migration. HNSCC subtypes, including OSCC, are characterized by a marked propensity for local invasion and lymphatic metastasis. Understanding the molecular mechanisms that mediate tumor invasion and metastasis may enable identification of novel therapeutic targets for management of tumor dissemination. Our study showed that knocked-down *SOX4* suppressed tumor cell invasion and migration in HNSCC cells; earlier studies showed it to significantly inhibit invasiveness and migration in prostate cancer cells [7]. These results indicate that SOX4 contributes to tumor progression and metastasis, and imply that SOX4 could be a useful target in cancer therapy.

Finally, SOX4 may serve as a biomarker for poor treatment response and outcome in OSCC. In this study, we found that SOX4 expression was significantly associated with recurrence, treatment failure and shorter OS. These results support our results of *in vitro* study, which associated SOX4 expression with oncogenic HNSCC phenotypes. Although several studies have associated SOX4 expression with shorter survival in prostate cancer, gastric cancer, and colon cancer [7, 9, 10], this is the first to demonstrate the correlation between SOX4 expression and treatment failure in OSCC. More accurate prediction of treatment failure would facilitate earlier recurrence detection and maximize the therapeutic effects of salvage treatment. In particular, among patients with OSCC who received chemotherapy and/or radiotherapy, those with high SOX4 expression had significantly shorter OS. These findings indicate that SOX4-related chemoradioresistance has a pessimistic effect on survival in patients with OSCC.

## Conclusions

Taken together, SOX4 may contribute to invasive and oncogenic phenotypes of HNSCC cells by promoting cell survival and causing chemoradioresistance. SOX4 may be a prognostic marker for OSCC survival outcomes and treatment response.

## Additional file

Additional file 1: **The extracted RNA loading was performed to verify the RNA integrity and double band (18S and 28S) was detected.** (JPEG 27 kb)

## Abbreviations

BSA: Bovine serum albumin
CRT: Chemoradiotherapy
DSS: Diease specific survival
GAPDH: Glyceraldehyde 3-phosphate dehydrogenase
HMG: High-mobility-group
HNSCC: Head and neck squamous cell carcinoma
OS: Overall survival
OSCC: Oral squamous cell carcinoma
PARP: Poly-ADP ribose polymerase
PBS: Phosphate buffered saline
RT-PCR: Reverse transcription-polymerase chain reaction
siRNA: Small interfering RNA
SOX: Sex-determining region Y related high-mobility-group box
*SOX4*-KO: SOX4 knockdown
SPSS: Statistical Package for the Social Sciences
SRY: Sex-determining region Y
XIAP: X-linked inhibitor of apoptosis protein
