# Supplementary figures and images for: SOX4 expression is associated with treatment failure and chemoradioresistance in oral squamous cell carcinoma
Source: BMC Cancer. 2015 Nov 10;15:888. doi: 10.1186/s12885-015-1875-8 (PMC4641419; doi:10.1186/s12885-015-1875-8)

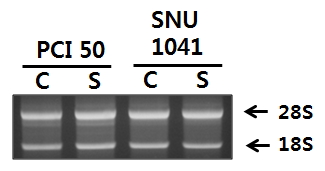

Supplement: Additional file 1: — The extracted RNA loading was performed to verify the RNA integrity and double band (18S and 28S) was detected. (JPEG 27 kb) [file 12885_2015_1875_MOESM1_ESM.jpeg]
